# 1-(Ferrocen-1-ylmeth­yl)-3-methyl­imidazol-3-ium hexa­fluorido­phosphate

**DOI:** 10.1107/S1600536812008719

**Published:** 2012-03-03

**Authors:** Vincent O. Nyamori, Siphesihle M. Zulu, Bernard Omondi

**Affiliations:** a School of Chemistry, University of KwaZulu-Natal, Westville Campus, Private Bag X54001, Durban 4000, South Africa

## Abstract

The crystal structure of the title compound, [Fe(C_5_H_5_)(C_10_H_12_N_2_)]PF_6_, consists of a ferrocene-1-methyl-(3-methyl­imidazolium) cation and a hexa­fluorido­phosphate anion. The ferrocenyl rings are skewed by 6.7 (4)° from the ideal eclipsed conformation. The inter­planar angle between the plane of the substituted cyclo­penta­dienyl ring and that of the imidazole ring is 89.9 (4)°. The crystal packing is stabilized by C—H⋯F hydrogen bonds.

## Related literature

For background to the chemistry of ferrocenes and their potential applications, see: Štěpnička (2008 ▶), Kealy & Pauson (1951 ▶); Togni & Hayashi (1995 ▶). For related work based on ferrocenylimidazolium salts, see: Nyamori *et al.* (2010*a* ▶); Thomas *et al.* (2000 ▶, 2002 ▶). For the synthesis, see: Nyamori *et al.* (2010*b* ▶). For related structures, see Nyamori & Bala (2008 ▶); Nyamori *et al.* (2010*a* ▶).
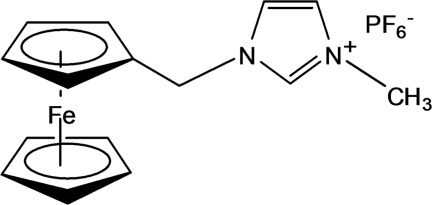


## Experimental

### 

#### Crystal data

[Fe(C_5_H_5_)(C_10_H_12_N_2_)]PF_6_
*M* *_r_* = 426.13
Orthorhombic, 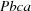

*a* = 12.4226 (2) Å
*b* = 13.4414 (2) Å
*c* = 19.2137 (3) Å
*V* = 3208.25 (9) Å^3^
*Z* = 8
Mo *K*α radiation
μ = 1.11 mm^−1^
*T* = 100 K
0.27 × 0.17 × 0.11 mm

#### Data collection

Bruker SMART APEXII CCD diffractometer
Absorption correction: multi-scan (*SADABS*; Bruker, 2008 ▶) *T*_min_ = 0.755, *T*_max_ = 0.888
44208 measured reflections
4041 independent reflections
3747 reflections with *I* > 2σ(*I*)
*R*_int_ = 0.029

#### Refinement

*R*[*F* ^2^ > 2σ(*F* ^2^)] = 0.024
*wR*(*F* ^2^) = 0.064
*S* = 1.04
4041 reflections
227 parameters
H-atom parameters constrained
Δρ_max_ = 0.46 e Å^−3^
Δρ_min_ = −0.36 e Å^−3^

### 

Data collection: *APEX2* (Bruker, 2008 ▶); cell refinement: *SAINT-Plus* (Bruker, 2008 ▶); data reduction: *SAINT-Plus* and *XPREP* (Bruker, 2008 ▶); program(s) used to solve structure: *SHELXS97* (Sheldrick, 2008 ▶); program(s) used to refine structure: *SHELXL97* (Sheldrick, 2008 ▶); molecular graphics: *ORTEP-3* (Farrugia, 1997 ▶); software used to prepare material for publication: *WinGX* (Farrugia, 1999 ▶).

## Supplementary Material

Crystal structure: contains datablock(s) global, I. DOI: 10.1107/S1600536812008719/fj2522sup1.cif

Structure factors: contains datablock(s) I. DOI: 10.1107/S1600536812008719/fj2522Isup2.hkl

Additional supplementary materials:  crystallographic information; 3D view; checkCIF report

## Figures and Tables

**Table 1:** Hydrogen-bond geometry (Å, °)

*D*—H⋯*A*	*D*—H	H⋯*A*	*D*⋯*A*	*D*—H⋯*A*
C14—H14⋯F1^i^	0.95	2.54	3.3249 (15)	140

Symmetry code: (i) 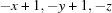.

**Fig. 1.**
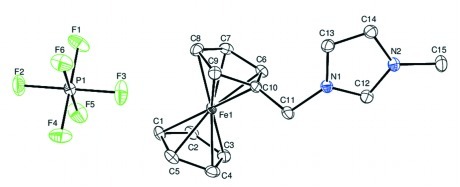
The asymmetric unit of the title compound with displacement ellipsoids drawn at the 50% probability level.

## supplementary crystallographic information

### Comment

The discovery of ferrocene heralded a new era in the realm of organometallic chemistry (Kealy & Pauson, 1951). The ferrocene group has unique electronic properties, such as ability to stabilize carbocations. The titled compound (I) consists of a ferrocenyl moiety linked to an imidazole group *via* a methylene group. The electronic system is very well conjugated and the compound exhibits resonance structures if in solution. The ferrocenyl moiety represents a quite bulky group with unique spatial requirements due to its sandwich shape, and electronically, the powerful donor capacity of ferrocene is important in the stabilization of highly reactive metal centres and other electroactive species. Some of the important properties that ferrocenyl containing imidazolium salts exhibit that makes their study significant include electronic stabilization of adjacent electron-deficient centres due to participation of the iron atom in the dispersal of the positive charge; the unique steric bulk, chemical stability and reversibility of the ferrocene/ferrocenium redox couple.

The ferrocenyl rings exhibit an eclipsed conformation with a significant staggering angle of 6.7° which is smaller than that of Nyamori & Bala (2008) however, Nyamori *et al.*, (2010*a*) have also synthesized ferrocenyl moiety with a significantly small staggering angle. The interplanar angle between the plane of the substituted Cp ring and that of the imidazole ring is orthogonal (89.9 (4)°) (Fig 1). In the crystal, a weak C–H···F hydrogen bond (Table 1) connects the cations and the anions.

### Experimental

In a two-neck round-bottom flask was added sodium hexafluoridophosphate (0.13 g, 0.76 mmol) and 1-(ferrocenylmethyl)-3-methylimidazolium iodide (0.30 g, 0.74 mmol) in acetone (20 ml). The mixture was stirred under a nitrogen atmosphere for 24 h at room temperature. The reaction mixture was filtered through a plug of celite and the filtrate was then concentrated *in vacuo* to yield 0.23 g, 72% of an orange crystals identified as 1-(Ferrocenylmethyl)-3-methylimidazole hexafluoridophosphate; mp 66–68 °C; IR (ATR cm_1) 3429, 1624, 1567, 1331, 1150, 812, 619,554, 500, 480; 1H NMR (CDCl3) 9.05 (1*H*, s, NCH), 7.11 (1*H*, s, NCH), 7.0868 (1*H*, s, NCH), 5.22 (2*H*, s, CH2), 4.39 (2*H*, t, J 1.8, C5H4), 4.23 (7*H*, t, J 1.8, C5H4), 4.24 (5*H*, s, C5H5), 3.94 (3*H*, s, CH3); 13 C NMR (CDCl3) 136.06, 122.79, 121.23, 78.02, 77.23, 69.97, 69.59, 69.19, 50.18; m/*z* 197 (2.3%), 198.6 (100%), 199.3 (13.1%), 280.6 (M±PF6, 4.5%); Anal. Calc. for C15H17N2Fe+; [*M*+]-PF6, 281.07411.

### Crystal data

**Table:**

[Fe(C_5_H_5_)(C_10_H_12_N_2_)]PF_6_	*F*(000) = 1728
*M**_r_* = 426.13	*D*_x_ = 1.764 Mg m^−^^3^
Orthorhombic, *P**b**c**a*	Mo *K*α radiation, λ = 0.71073 Å
Hall symbol: -P 2ac 2ab	Cell parameters from 47745 reflections
*a* = 12.4226 (2) Å	θ = 2.1–28.6°
*b* = 13.4414 (2) Å	µ = 1.11 mm^−^^1^
*c* = 19.2137 (3) Å	*T* = 100 K
*V* = 3208.25 (9) Å^3^	Block, yellow
*Z* = 8	0.27 × 0.17 × 0.11 mm

### Data collection

**Table:**

Bruker SMART APEXII CCD diffractometer	3747 reflections with *I* > 2σ(*I*)
Graphite monochromator	*R*_int_ = 0.029
φ and ω scans	θ_max_ = 28.6°, θ_min_ = 2.1°
Absorption correction: multi-scan (*SADABS*; Bruker, 2008)	*h* = −16→16
*T*_min_ = 0.755, *T*_max_ = 0.888	*k* = −18→17
44208 measured reflections	*l* = −25→25
4041 independent reflections	

### Refinement

**Table:**

Refinement on *F*^2^	Primary atom site location: structure-invariant direct methods
Least-squares matrix: full	Secondary atom site location: difference Fourier map
*R*[*F*^2^ > 2σ(*F*^2^)] = 0.024	Hydrogen site location: inferred from neighbouring sites
*wR*(*F*^2^) = 0.064	H-atom parameters constrained
*S* = 1.04	*w* = 1/[σ^2^(*F*_o_^2^) + (0.0307*P*)^2^ + 2.2775*P*] where *P* = (*F*_o_^2^ + 2*F*_c_^2^)/3
4041 reflections	(Δ/σ)_max_ = 0.001
227 parameters	Δρ_max_ = 0.46 e Å^−^^3^
0 restraints	Δρ_min_ = −0.36 e Å^−^^3^

### Special details

**Table:**

Experimental. Carbon-bound H-atoms were placed in calculated positions [C—H = 0.98 Å for Me H atoms, 0.99 Å for Methylene H atoms, 0.99 for methine H atoms and 0.95 Å for aromatic H atoms; *U*_iso_(H) = 1.2*U*_eq_(C) (1.5 for Me groups)] and were included in the refinement in the riding model approximation. The N—H H-atom was located in a difference map and freely refined with N—H = 0.88 Å (*U*_iso_(H) = 1.2*U*_eq_(N).
Geometry. All e.s.d.'s (except the e.s.d. in the dihedral angle between two l.s. planes) are estimated using the full covariance matrix. The cell e.s.d.'s are taken into account individually in the estimation of e.s.d.'s in distances, angles and torsion angles; correlations between e.s.d.'s in cell parameters are only used when they are defined by crystal symmetry. An approximate (isotropic) treatment of cell e.s.d.'s is used for estimating e.s.d.'s involving l.s. planes.
Refinement. Refinement of *F*^2^ against ALL reflections. The weighted *R*-factor *wR* and goodness of fit *S* are based on *F*^2^, conventional *R*-factors *R* are based on *F*, with *F* set to zero for negative *F*^2^. The threshold expression of *F*^2^ > σ(*F*^2^) is used only for calculating *R*-factors(gt) *etc*. and is not relevant to the choice of reflections for refinement. *R*-factors based on *F*^2^ are statistically about twice as large as those based on *F*, and *R*- factors based on ALL data will be even larger. The following ALERTS were generated. Each ALERT has the format test-name_ALERT_alert-type_alert-level. PLAT244_ALERT_4_C Low 'Solvent' *U*_eq_ as Compared to Neighbors of P1 PLAT912_ALERT_4_C Missing # of FCF Reflections Above STh/*L*= 0.600 62 PLAT960_ALERT_3_G Number of Intensities with *I*. LT. - 2*sig(*I*) ··· 4 Noted:

### Fractional atomic coordinates and isotropic or equivalent isotropic displacement parameters (Å^2^)

**Table:**

	*x*	*y*	*z*	*U*_iso_*/*U*_eq_	
C1	0.70208 (10)	0.06956 (9)	0.08674 (7)	0.0187 (2)	
H1	0.6918	0.0677	0.1383	0.022*	
C2	0.63898 (10)	0.01756 (9)	0.03683 (7)	0.0190 (2)	
H2	0.5772	−0.0278	0.0471	0.023*	
C3	0.67965 (11)	0.04158 (10)	−0.03054 (7)	0.0216 (3)	
H3	0.6515	0.0158	−0.0758	0.026*	
C4	0.76762 (11)	0.10892 (10)	−0.02199 (7)	0.0219 (3)	
H4	0.8118	0.1387	−0.0602	0.026*	
C5	0.78092 (10)	0.12621 (10)	0.05054 (7)	0.0203 (3)	
H5	0.8359	0.1708	0.0723	0.024*	
C6	0.50453 (10)	0.22435 (9)	−0.03703 (7)	0.0163 (2)	
H6	0.4707	0.1933	−0.079	0.02*	
C7	0.47250 (10)	0.20950 (9)	0.03352 (7)	0.0183 (2)	
H7	0.4127	0.1654	0.0497	0.022*	
C8	0.54191 (11)	0.26670 (9)	0.07702 (7)	0.0189 (2)	
H8	0.5392	0.2697	0.129	0.023*	
C9	0.61633 (11)	0.31834 (9)	0.03367 (7)	0.0168 (2)	
H9	0.6748	0.364	0.0498	0.02*	
C10	0.59352 (10)	0.29181 (9)	−0.03694 (6)	0.0144 (2)	
C11	0.65066 (11)	0.32967 (9)	−0.10021 (7)	0.0178 (2)	
H11A	0.7149	0.3681	−0.0859	0.021*	
H11B	0.675	0.2728	−0.1289	0.021*	
C12	0.55414 (10)	0.37912 (9)	−0.20841 (6)	0.0165 (2)	
H12	0.5825	0.328	−0.2372	0.02*	
C13	0.52134 (10)	0.47496 (9)	−0.11803 (7)	0.0174 (2)	
H13	0.5234	0.502	−0.0724	0.021*	
C14	0.46165 (10)	0.50875 (9)	−0.17209 (7)	0.0174 (2)	
H14	0.4139	0.5639	−0.1716	0.021*	
C15	0.43846 (11)	0.45908 (10)	−0.29794 (7)	0.0213 (3)	
H15A	0.4663	0.5202	−0.3191	0.032*	
H15B	0.3598	0.4628	−0.295	0.032*	
H15C	0.4591	0.4018	−0.3265	0.032*	
N1	0.57853 (8)	0.39380 (8)	−0.14182 (5)	0.0149 (2)	
N2	0.48347 (9)	0.44783 (8)	−0.22797 (5)	0.0152 (2)	
F1	0.68267 (7)	0.32029 (6)	0.25538 (6)	0.0312 (2)	
F2	0.83765 (8)	0.29514 (8)	0.31564 (5)	0.0337 (2)	
F3	0.78054 (9)	0.34815 (8)	0.15824 (5)	0.0390 (2)	
F4	0.93499 (7)	0.32338 (7)	0.21887 (5)	0.0332 (2)	
F5	0.80333 (8)	0.20561 (7)	0.21913 (5)	0.0346 (2)	
F6	0.81291 (7)	0.43793 (6)	0.25467 (5)	0.0303 (2)	
Fe1	0.629560 (14)	0.167551 (12)	0.019064 (9)	0.01225 (6)	
P1	0.80910 (3)	0.32180 (2)	0.236862 (17)	0.01482 (8)	

### Atomic displacement parameters (Å^2^)

**Table:**

	*U*^11^	*U*^22^	*U*^33^	*U*^12^	*U*^13^	*U*^23^
C1	0.0200 (6)	0.0167 (6)	0.0193 (6)	0.0016 (5)	−0.0043 (5)	0.0048 (5)
C2	0.0192 (6)	0.0114 (5)	0.0265 (6)	0.0006 (4)	−0.0035 (5)	0.0019 (5)
C3	0.0267 (7)	0.0166 (6)	0.0216 (6)	0.0081 (5)	−0.0035 (5)	−0.0036 (5)
C4	0.0201 (6)	0.0204 (6)	0.0252 (7)	0.0075 (5)	0.0046 (5)	0.0053 (5)
C5	0.0140 (5)	0.0189 (6)	0.0280 (7)	0.0006 (5)	−0.0047 (5)	0.0047 (5)
C6	0.0148 (5)	0.0145 (5)	0.0196 (6)	0.0021 (4)	−0.0032 (4)	0.0010 (5)
C7	0.0153 (5)	0.0164 (6)	0.0233 (6)	0.0028 (5)	0.0022 (5)	0.0026 (5)
C8	0.0243 (6)	0.0157 (5)	0.0167 (6)	0.0036 (5)	0.0026 (5)	−0.0008 (4)
C9	0.0211 (6)	0.0120 (5)	0.0172 (6)	0.0009 (4)	−0.0013 (5)	−0.0012 (4)
C10	0.0157 (5)	0.0120 (5)	0.0155 (5)	0.0026 (4)	−0.0007 (4)	0.0014 (4)
C11	0.0171 (5)	0.0189 (6)	0.0174 (6)	0.0043 (5)	0.0003 (5)	0.0053 (4)
C12	0.0188 (6)	0.0138 (5)	0.0168 (6)	−0.0001 (4)	0.0000 (4)	0.0001 (4)
C13	0.0209 (6)	0.0155 (5)	0.0159 (6)	0.0027 (5)	0.0016 (5)	0.0002 (4)
C14	0.0192 (6)	0.0149 (5)	0.0182 (6)	0.0017 (5)	0.0014 (5)	0.0009 (4)
C15	0.0263 (7)	0.0217 (6)	0.0158 (6)	0.0013 (5)	−0.0058 (5)	0.0009 (5)
N1	0.0161 (5)	0.0134 (5)	0.0154 (5)	0.0011 (4)	0.0005 (4)	0.0028 (4)
N2	0.0165 (5)	0.0140 (5)	0.0152 (5)	−0.0012 (4)	−0.0017 (4)	0.0010 (4)
F1	0.0143 (4)	0.0267 (4)	0.0526 (6)	−0.0019 (3)	−0.0009 (4)	0.0108 (4)
F2	0.0334 (5)	0.0497 (6)	0.0180 (4)	−0.0015 (4)	−0.0067 (4)	0.0080 (4)
F3	0.0562 (6)	0.0405 (5)	0.0202 (4)	−0.0019 (5)	−0.0115 (4)	0.0082 (4)
F4	0.0205 (4)	0.0331 (5)	0.0459 (6)	0.0019 (4)	0.0112 (4)	0.0017 (4)
F5	0.0383 (5)	0.0165 (4)	0.0490 (6)	0.0017 (4)	−0.0128 (4)	−0.0042 (4)
F6	0.0241 (4)	0.0188 (4)	0.0481 (6)	−0.0028 (3)	−0.0019 (4)	−0.0082 (4)
Fe1	0.01293 (10)	0.01083 (9)	0.01299 (9)	0.00042 (6)	−0.00162 (6)	0.00074 (6)
P1	0.01470 (15)	0.01456 (14)	0.01521 (15)	−0.00112 (11)	−0.00222 (11)	0.00146 (11)

### Geometric parameters (Å, º)

**Table:**

C1—C2	1.4222 (18)	C9—Fe1	2.0527 (12)
C1—C5	1.4223 (18)	C9—H9	1
C1—Fe1	2.0585 (12)	C10—C11	1.4968 (17)
C1—H1	1	C10—Fe1	2.0367 (12)
C2—C3	1.4267 (19)	C11—N1	1.4782 (15)
C2—Fe1	2.0482 (13)	C11—H11A	0.99
C2—H2	1	C11—H11B	0.99
C3—C4	1.428 (2)	C12—N2	1.3285 (16)
C3—Fe1	2.0403 (13)	C12—N1	1.3296 (16)
C3—H3	1	C12—H12	0.95
C4—C5	1.4225 (19)	C13—C14	1.3547 (17)
C4—Fe1	2.0456 (13)	C13—N1	1.3797 (16)
C4—H4	1	C13—H13	0.95
C5—Fe1	2.0519 (13)	C14—N2	1.3772 (16)
C5—H5	1	C14—H14	0.95
C6—C7	1.4269 (18)	C15—N2	1.4637 (16)
C6—C10	1.4299 (17)	C15—H15A	0.98
C6—Fe1	2.0389 (12)	C15—H15B	0.98
C6—H6	1	C15—H15C	0.98
C7—C8	1.4258 (19)	F1—P1	1.6106 (9)
C7—Fe1	2.0498 (13)	F2—P1	1.5954 (9)
C7—H7	1	F3—P1	1.5917 (9)
C8—C9	1.4249 (18)	F4—P1	1.6018 (9)
C8—Fe1	2.0497 (13)	F5—P1	1.6001 (9)
C8—H8	1	F6—P1	1.5988 (9)
C9—C10	1.4310 (17)		
			
C2—C1—C5	108.23 (12)	N2—C14—H14	126.6
C2—C1—Fe1	69.35 (7)	N2—C15—H15A	109.5
C5—C1—Fe1	69.50 (7)	N2—C15—H15B	109.5
C2—C1—H1	125.9	H15A—C15—H15B	109.5
C5—C1—H1	125.9	N2—C15—H15C	109.5
Fe1—C1—H1	125.9	H15A—C15—H15C	109.5
C1—C2—C3	107.78 (12)	H15B—C15—H15C	109.5
C1—C2—Fe1	70.13 (7)	C12—N1—C13	108.59 (10)
C3—C2—Fe1	69.28 (7)	C12—N1—C11	124.89 (11)
C1—C2—H2	126.1	C13—N1—C11	126.45 (11)
C3—C2—H2	126.1	C12—N2—C14	108.84 (10)
Fe1—C2—H2	126.1	C12—N2—C15	125.74 (11)
C2—C3—C4	108.06 (12)	C14—N2—C15	125.40 (11)
C2—C3—Fe1	69.87 (7)	C10—Fe1—C6	41.08 (5)
C4—C3—Fe1	69.74 (8)	C10—Fe1—C3	120.05 (5)
C2—C3—H3	126	C6—Fe1—C3	107.22 (5)
C4—C3—H3	126	C10—Fe1—C4	107.24 (5)
Fe1—C3—H3	126	C6—Fe1—C4	125.38 (5)
C5—C4—C3	107.76 (12)	C3—Fe1—C4	40.93 (6)
C5—C4—Fe1	69.92 (7)	C10—Fe1—C2	155.21 (5)
C3—C4—Fe1	69.34 (8)	C6—Fe1—C2	120.01 (5)
C5—C4—H4	126.1	C3—Fe1—C2	40.85 (5)
C3—C4—H4	126.1	C4—Fe1—C2	68.73 (5)
Fe1—C4—H4	126.1	C10—Fe1—C8	68.72 (5)
C1—C5—C4	108.16 (12)	C6—Fe1—C8	68.84 (5)
C1—C5—Fe1	70.01 (7)	C3—Fe1—C8	162.82 (6)
C4—C5—Fe1	69.45 (7)	C4—Fe1—C8	154.87 (6)
C1—C5—H5	125.9	C2—Fe1—C8	125.44 (5)
C4—C5—H5	125.9	C10—Fe1—C7	68.70 (5)
Fe1—C5—H5	125.9	C6—Fe1—C7	40.85 (5)
C7—C6—C10	107.64 (11)	C3—Fe1—C7	125.58 (6)
C7—C6—Fe1	69.99 (7)	C4—Fe1—C7	162.98 (6)
C10—C6—Fe1	69.38 (7)	C2—Fe1—C7	107.61 (5)
C7—C6—H6	126.2	C8—Fe1—C7	40.70 (5)
C10—C6—H6	126.2	C10—Fe1—C5	125.40 (5)
Fe1—C6—H6	126.2	C6—Fe1—C5	162.81 (5)
C8—C7—C6	108.23 (11)	C3—Fe1—C5	68.50 (6)
C8—C7—Fe1	69.65 (7)	C4—Fe1—C5	40.63 (5)
C6—C7—Fe1	69.17 (7)	C2—Fe1—C5	68.40 (5)
C8—C7—H7	125.9	C8—Fe1—C5	120.18 (6)
C6—C7—H7	125.9	C7—Fe1—C5	155.07 (6)
Fe1—C7—H7	125.9	C10—Fe1—C9	40.97 (5)
C9—C8—C7	108.20 (11)	C6—Fe1—C9	69.00 (5)
C9—C8—Fe1	69.79 (7)	C3—Fe1—C9	155.19 (6)
C7—C8—Fe1	69.65 (7)	C4—Fe1—C9	120.01 (6)
C9—C8—H8	125.9	C2—Fe1—C9	162.50 (6)
C7—C8—H8	125.9	C8—Fe1—C9	40.65 (5)
Fe1—C8—H8	125.9	C7—Fe1—C9	68.51 (5)
C8—C9—C10	107.72 (11)	C5—Fe1—C9	107.49 (5)
C8—C9—Fe1	69.57 (7)	C10—Fe1—C1	162.69 (5)
C10—C9—Fe1	68.92 (7)	C6—Fe1—C1	155.08 (5)
C8—C9—H9	126.1	C3—Fe1—C1	68.32 (5)
C10—C9—H9	126.1	C4—Fe1—C1	68.30 (5)
Fe1—C9—H9	126.1	C2—Fe1—C1	40.52 (5)
C6—C10—C9	108.20 (11)	C8—Fe1—C1	107.78 (5)
C6—C10—C11	125.54 (11)	C7—Fe1—C1	120.46 (5)
C9—C10—C11	126.25 (11)	C5—Fe1—C1	40.49 (5)
C6—C10—Fe1	69.54 (7)	C9—Fe1—C1	125.48 (5)
C9—C10—Fe1	70.12 (7)	F3—P1—F2	179.87 (7)
C11—C10—Fe1	127.13 (9)	F3—P1—F6	89.57 (6)
N1—C11—C10	110.49 (10)	F2—P1—F6	90.55 (6)
N1—C11—H11A	109.6	F3—P1—F5	90.29 (6)
C10—C11—H11A	109.6	F2—P1—F5	89.58 (6)
N1—C11—H11B	109.6	F6—P1—F5	179.13 (5)
C10—C11—H11B	109.6	F3—P1—F4	90.57 (6)
H11A—C11—H11B	108.1	F2—P1—F4	89.46 (5)
N2—C12—N1	108.65 (11)	F6—P1—F4	90.25 (5)
N2—C12—H12	125.7	F5—P1—F4	90.62 (5)
N1—C12—H12	125.7	F3—P1—F1	89.71 (6)
C14—C13—N1	107.04 (11)	F2—P1—F1	90.25 (5)
C14—C13—H13	126.5	F6—P1—F1	89.65 (5)
N1—C13—H13	126.5	F5—P1—F1	89.49 (5)
C13—C14—N2	106.90 (11)	F4—P1—F1	179.70 (6)
C13—C14—H14	126.6		
			
C5—C1—C2—C3	0.48 (14)	C5—C4—Fe1—C3	−119.01 (11)
Fe1—C1—C2—C3	59.27 (9)	C5—C4—Fe1—C2	−81.21 (8)
C5—C1—C2—Fe1	−58.79 (9)	C3—C4—Fe1—C2	37.80 (8)
C1—C2—C3—C4	−0.30 (15)	C5—C4—Fe1—C8	48.21 (15)
Fe1—C2—C3—C4	59.51 (9)	C3—C4—Fe1—C8	167.22 (11)
C1—C2—C3—Fe1	−59.80 (9)	C5—C4—Fe1—C7	−161.85 (16)
C2—C3—C4—C5	0.00 (15)	C3—C4—Fe1—C7	−42.8 (2)
Fe1—C3—C4—C5	59.60 (9)	C3—C4—Fe1—C5	119.01 (11)
C2—C3—C4—Fe1	−59.59 (9)	C5—C4—Fe1—C9	81.93 (9)
C2—C1—C5—C4	−0.48 (15)	C3—C4—Fe1—C9	−159.06 (8)
Fe1—C1—C5—C4	−59.17 (9)	C5—C4—Fe1—C1	−37.51 (8)
C2—C1—C5—Fe1	58.70 (9)	C3—C4—Fe1—C1	81.50 (8)
C3—C4—C5—C1	0.29 (15)	C1—C2—Fe1—C10	166.23 (11)
Fe1—C4—C5—C1	59.52 (9)	C3—C2—Fe1—C10	47.29 (15)
C3—C4—C5—Fe1	−59.23 (9)	C1—C2—Fe1—C6	−159.41 (8)
C10—C6—C7—C8	−0.54 (14)	C3—C2—Fe1—C6	81.65 (9)
Fe1—C6—C7—C8	58.86 (9)	C1—C2—Fe1—C3	118.94 (11)
C10—C6—C7—Fe1	−59.39 (8)	C1—C2—Fe1—C4	81.07 (8)
C6—C7—C8—C9	0.78 (14)	C3—C2—Fe1—C4	−37.87 (8)
Fe1—C7—C8—C9	59.34 (9)	C1—C2—Fe1—C8	−75.18 (9)
C6—C7—C8—Fe1	−58.56 (9)	C3—C2—Fe1—C8	165.88 (8)
C7—C8—C9—C10	−0.73 (14)	C1—C2—Fe1—C7	−116.57 (8)
Fe1—C8—C9—C10	58.53 (8)	C3—C2—Fe1—C7	124.49 (8)
C7—C8—C9—Fe1	−59.26 (9)	C1—C2—Fe1—C5	37.27 (8)
C7—C6—C10—C9	0.09 (14)	C3—C2—Fe1—C5	−81.67 (8)
Fe1—C6—C10—C9	−59.69 (8)	C1—C2—Fe1—C9	−42.3 (2)
C7—C6—C10—C11	−178.54 (11)	C3—C2—Fe1—C9	−161.25 (16)
Fe1—C6—C10—C11	121.69 (12)	C3—C2—Fe1—C1	−118.94 (11)
C7—C6—C10—Fe1	59.77 (8)	C9—C8—Fe1—C10	−37.78 (7)
C8—C9—C10—C6	0.39 (14)	C7—C8—Fe1—C10	81.67 (8)
Fe1—C9—C10—C6	59.33 (8)	C9—C8—Fe1—C6	−82.00 (8)
C8—C9—C10—C11	179.01 (11)	C7—C8—Fe1—C6	37.45 (7)
Fe1—C9—C10—C11	−122.06 (12)	C9—C8—Fe1—C3	−161.81 (16)
C8—C9—C10—Fe1	−58.94 (9)	C7—C8—Fe1—C3	−42.4 (2)
C6—C10—C11—N1	66.58 (15)	C9—C8—Fe1—C4	47.57 (15)
C9—C10—C11—N1	−111.80 (14)	C7—C8—Fe1—C4	167.01 (11)
Fe1—C10—C11—N1	156.70 (9)	C9—C8—Fe1—C2	165.49 (8)
N1—C13—C14—N2	−0.09 (14)	C7—C8—Fe1—C2	−75.06 (9)
N2—C12—N1—C13	−0.05 (14)	C9—C8—Fe1—C7	−119.45 (11)
N2—C12—N1—C11	176.98 (11)	C9—C8—Fe1—C5	81.73 (9)
C14—C13—N1—C12	0.09 (14)	C7—C8—Fe1—C5	−158.82 (7)
C14—C13—N1—C11	−176.88 (11)	C7—C8—Fe1—C9	119.45 (11)
C10—C11—N1—C12	−123.38 (13)	C9—C8—Fe1—C1	124.22 (8)
C10—C11—N1—C13	53.11 (16)	C7—C8—Fe1—C1	−116.34 (8)
N1—C12—N2—C14	−0.01 (14)	C8—C7—Fe1—C10	−81.71 (8)
N1—C12—N2—C15	178.28 (11)	C6—C7—Fe1—C10	38.17 (7)
C13—C14—N2—C12	0.06 (14)	C8—C7—Fe1—C6	−119.88 (11)
C13—C14—N2—C15	−178.23 (12)	C8—C7—Fe1—C3	165.83 (8)
C9—C10—Fe1—C6	119.29 (11)	C6—C7—Fe1—C3	−74.28 (9)
C11—C10—Fe1—C6	−119.72 (14)	C8—C7—Fe1—C4	−160.96 (17)
C6—C10—Fe1—C3	81.77 (9)	C6—C7—Fe1—C4	−41.1 (2)
C9—C10—Fe1—C3	−158.93 (8)	C8—C7—Fe1—C2	124.32 (8)
C11—C10—Fe1—C3	−37.94 (13)	C6—C7—Fe1—C2	−115.80 (8)
C6—C10—Fe1—C4	124.51 (8)	C6—C7—Fe1—C8	119.88 (11)
C9—C10—Fe1—C4	−116.19 (8)	C8—C7—Fe1—C5	47.80 (15)
C11—C10—Fe1—C4	4.80 (12)	C6—C7—Fe1—C5	167.69 (11)
C6—C10—Fe1—C2	48.05 (15)	C8—C7—Fe1—C9	−37.56 (7)
C9—C10—Fe1—C2	167.34 (12)	C6—C7—Fe1—C9	82.32 (8)
C11—C10—Fe1—C2	−71.67 (17)	C8—C7—Fe1—C1	81.92 (9)
C6—C10—Fe1—C8	−81.80 (8)	C6—C7—Fe1—C1	−158.19 (7)
C9—C10—Fe1—C8	37.50 (8)	C1—C5—Fe1—C10	166.27 (7)
C11—C10—Fe1—C8	158.48 (13)	C4—C5—Fe1—C10	−74.35 (9)
C6—C10—Fe1—C7	−37.97 (7)	C1—C5—Fe1—C6	−160.03 (16)
C9—C10—Fe1—C7	81.33 (8)	C4—C5—Fe1—C6	−40.7 (2)
C11—C10—Fe1—C7	−157.68 (12)	C1—C5—Fe1—C3	−81.37 (8)
C6—C10—Fe1—C5	165.55 (8)	C4—C5—Fe1—C3	38.01 (8)
C9—C10—Fe1—C5	−75.16 (9)	C1—C5—Fe1—C4	−119.38 (11)
C11—C10—Fe1—C5	45.83 (13)	C1—C5—Fe1—C2	−37.30 (8)
C6—C10—Fe1—C9	−119.29 (11)	C4—C5—Fe1—C2	82.08 (9)
C11—C10—Fe1—C9	120.99 (14)	C1—C5—Fe1—C8	82.11 (9)
C6—C10—Fe1—C1	−163.26 (16)	C4—C5—Fe1—C8	−158.51 (8)
C9—C10—Fe1—C1	−44.0 (2)	C1—C5—Fe1—C7	48.13 (16)
C11—C10—Fe1—C1	77.0 (2)	C4—C5—Fe1—C7	167.51 (11)
C7—C6—Fe1—C10	−118.80 (10)	C1—C5—Fe1—C9	124.63 (8)
C7—C6—Fe1—C3	124.95 (8)	C4—C5—Fe1—C9	−115.99 (8)
C10—C6—Fe1—C3	−116.25 (8)	C4—C5—Fe1—C1	119.38 (11)
C7—C6—Fe1—C4	166.36 (8)	C8—C9—Fe1—C10	119.45 (11)
C10—C6—Fe1—C4	−74.85 (9)	C8—C9—Fe1—C6	81.59 (8)
C7—C6—Fe1—C2	82.31 (9)	C10—C9—Fe1—C6	−37.87 (7)
C10—C6—Fe1—C2	−158.90 (7)	C8—C9—Fe1—C3	167.31 (12)
C7—C6—Fe1—C8	−37.32 (7)	C10—C9—Fe1—C3	47.85 (16)
C10—C6—Fe1—C8	81.47 (8)	C8—C9—Fe1—C4	−158.77 (8)
C10—C6—Fe1—C7	118.80 (10)	C10—C9—Fe1—C4	81.77 (9)
C7—C6—Fe1—C5	−162.29 (16)	C8—C9—Fe1—C2	−42.8 (2)
C10—C6—Fe1—C5	−43.5 (2)	C10—C9—Fe1—C2	−162.21 (16)
C7—C6—Fe1—C9	−81.03 (8)	C10—C9—Fe1—C8	−119.45 (11)
C10—C6—Fe1—C9	37.77 (7)	C8—C9—Fe1—C7	37.61 (8)
C7—C6—Fe1—C1	49.47 (15)	C10—C9—Fe1—C7	−81.84 (8)
C10—C6—Fe1—C1	168.26 (11)	C8—C9—Fe1—C5	−116.25 (8)
C2—C3—Fe1—C10	−159.15 (7)	C10—C9—Fe1—C5	124.30 (8)
C4—C3—Fe1—C10	81.69 (9)	C8—C9—Fe1—C1	−75.24 (9)
C2—C3—Fe1—C6	−116.24 (8)	C10—C9—Fe1—C1	165.30 (7)
C4—C3—Fe1—C6	124.60 (8)	C2—C1—Fe1—C10	−160.41 (16)
C2—C3—Fe1—C4	119.16 (11)	C5—C1—Fe1—C10	−40.6 (2)
C4—C3—Fe1—C2	−119.16 (11)	C2—C1—Fe1—C6	46.28 (16)
C2—C3—Fe1—C8	−42.3 (2)	C5—C1—Fe1—C6	166.14 (11)
C4—C3—Fe1—C8	−161.45 (16)	C2—C1—Fe1—C3	−38.02 (8)
C2—C3—Fe1—C7	−75.00 (9)	C5—C1—Fe1—C3	81.84 (9)
C4—C3—Fe1—C7	165.84 (8)	C2—C1—Fe1—C4	−82.22 (9)
C2—C3—Fe1—C5	81.42 (8)	C5—C1—Fe1—C4	37.64 (8)
C4—C3—Fe1—C5	−37.74 (8)	C5—C1—Fe1—C2	119.86 (11)
C2—C3—Fe1—C9	166.68 (11)	C2—C1—Fe1—C8	124.19 (8)
C4—C3—Fe1—C9	47.53 (16)	C5—C1—Fe1—C8	−115.95 (8)
C2—C3—Fe1—C1	37.73 (8)	C2—C1—Fe1—C7	81.50 (9)
C4—C3—Fe1—C1	−81.43 (8)	C5—C1—Fe1—C7	−158.64 (8)
C5—C4—Fe1—C10	124.73 (8)	C2—C1—Fe1—C5	−119.86 (11)
C3—C4—Fe1—C10	−116.26 (8)	C2—C1—Fe1—C9	165.61 (8)
C5—C4—Fe1—C6	166.34 (7)	C5—C1—Fe1—C9	−74.54 (10)
C3—C4—Fe1—C6	−74.65 (9)		

### Hydrogen-bond geometry (Å, º)

**Table:**

*D*—H···*A*	*D*—H	H···*A*	*D*···*A*	*D*—H···*A*
C14—H14···F1^i^	0.95	2.54	3.3249 (15)	140

Symmetry code: (i) −*x*+1, −*y*+1, −*z*.
